# Effectiveness of Telerehabilitation in Dizziness: A Systematic Review with Meta-Analysis

**DOI:** 10.3390/s24103028

**Published:** 2024-05-10

**Authors:** Davide Grillo, Mirko Zitti, Błażej Cieślik, Stefano Vania, Silvia Zangarini, Stefano Bargellesi, Pawel Kiper

**Affiliations:** 1 Physical Medicine and Rehabilitation Unit, Azienda ULSS 3 Serenissima, 30126 Venice, Italy; davide.grillo@aulss3.veneto.it (D.G.); silvia.zangarini@aulss3.veneto.it (S.Z.); stefano.bargellesi@aulss3.veneto.it (S.B.); 2 Healthcare Innovation Technology Lab, IRCCS San Camillo Hospital, 30126 Venezia, Italy; mirko.zitti@hsancamillo.it (M.Z.);; 3 Department of Neuroscience, Rehabilitation, Ophthalmology, Genetics, Maternal and Child Health, University of Genova, Campus of Savona, 17100 Savona, Italy

**Keywords:** vertigo, remote support, physiotherapy, vestibular disorder, balance, depression, anxiety, telehealth

## Abstract

Dizziness can be a debilitating condition with various causes, with at least one episode reported in 17% to 30% of the international adult population. Given the effectiveness of rehabilitation in treating dizziness and the recent advancements in telerehabilitation, this systematic review aims to investigate the effectiveness of telerehabilitation in the treatment of this disorder. The search, conducted across Medline, Cochrane Central Register of Controlled Trials, and PEDro databases, included randomized controlled trials assessing the efficacy of telerehabilitation interventions, delivered synchronously, asynchronously, or via tele-support/monitoring. Primary outcomes focused on dizziness frequency/severity and disability, with secondary outcomes assessing anxiety and depression measures. Seven articles met the eligibility criteria, whereas five articles contributed to the meta-analysis. Significant findings were observed regarding the frequency and severity of dizziness (mean difference of 3.01, *p* < 0.001), disability (mean difference of −4.25, *p* < 0.001), and anxiety (standardized mean difference of −0.16, *p* = 0.02), favoring telerehabilitation. Telerehabilitation shows promise as a treatment for dizziness, aligning with the positive outcomes seen in traditional rehabilitation studies. However, the effectiveness of different telerehabilitation approaches requires further investigation, given the moderate methodological quality and the varied nature of existing methods and programs.

## 1. Introduction

To date, estimates indicate that between 17% and 30% of the global adult population has experienced at least one significant episode of dizziness, with an annual incidence of 1.4% and increasing healthcare expenditure due to the rising average age of the population [1]. The “*SHARE*” survey, conducted on over 69,000 individuals aged over 50 in twenty different European countries, has provided recent data on the prevalence among different states (with a total figure of 12.4% in 2017), as well as additional information on risk factors [2]. Furthermore, in order to facilitate access and exchange of available data, improve diagnostic and therapeutic standards, and foster multidisciplinary collaboration between countries, data collection registries for patients with dizziness are gradually emerging [1,2,3,4,5].

The term dizziness, which is the sensation of disturbed or impaired spatial orientation without a false or distorted sense of motion [6], has been chosen as an umbrella term encompassing all conditions that present with clinical manifestations of vertigo. In fact, vertigo should be considered as a more or less common symptom of various etiologies rather than stand-alone pathologies [7,8]. Historically, clinical presentations of patients with dizziness were commonly distinguished as follows: vertigo, the illusion of rotary movements or other false movements; pre syncope the sensation of imminent fainting or loss of consciousness; disequilibrium, or loss of balance without other altered sensations related to the head; undefined, i.e., other manifestations such as dizziness, lightheadedness, etc. [9].

However, over time, such differentiation proved to be limiting for a proper patient assessment [10]. With the advancement of technologies and research, more precise classifications in terms of diagnostic, epidemiological, and therapeutic criteria, as well as new patient management strategies, are now available and help us in the assessment and management of patients with dizziness [6]. Characteristic patterns of dizziness can arise from dysfunction of the vestibular system, both peripheral (benign paroxysmal positional vertigo, unilateral or bilateral vestibulopathy, Meniere’s syndrome, fistulas, etc.) and central (central vertigo due to cerebrovascular disorders, migraines, demyelination, etc.), sometimes classified as “non-specific dizziness” [11]. Functional dizziness includes psychogenic vertigo and “persistent postural-perception dizziness” (PPPD). Other forms include pharmacologically induced dizziness (as a side effect of anti-epileptic drugs), arterial hypotension, and cervicogenic vertigo [12,13]. Vestibular rehabilitation plays an important role in the treatment and management of dizziness in various etiological conditions. Studies demonstrate its effectiveness in cases of peripheral vestibular dysfunction [14], central vertigo [15], cervicogenic vertigo [16], and neurological disorders [17], as well as in adulthood [18], either as a stand-alone therapy or in combination with other therapeutic approaches [19]. The initial proposed exercises were those of Cawthorne–Cooksey, developed to treat patients with labyrinthine injuries from surgery or head trauma [20].

The evolution of research has progressively led to more effective interventions. Currently, vestibular rehabilitation utilizes exercises involving the visual system, head, and trunk, with the aim of stimulating the three subsystems responsible for maintaining balance: the visual, proprioceptive, and vestibular systems. Vestibular rehabilitation triggers responses in our organism through compensation, thanks to the plasticity of our central nervous system, in which, through movement, it habituates and reduces susceptibility to repetitive stimuli from vestibular nuclei response (a process often referred to as “habituation”); adaptation for vestibulo-visual interaction (gaze stabilization) and potentially also for eye–hand coordination, using provocative and repetitive head or eye movements to reduce error possibility and restore the vestibulo-ocular reflex (VOR); substitution, which promotes the use of individual combinations of sensory inputs (such as visual or sensorimotor) to compensate for dysfunctional vestibular inputs or reinforce relative compensation; and reconditioning of postural control and functional activities, based on the principles of motor learning to modify movement characteristics [21]. These exercises need to be adjusted and balanced according to the characteristics of the patient: it is necessary to structure a proper progression of the exercise in line with the patient’s capabilities and potential along the rehabilitation path, establishing an appropriate and challenging level of difficulty for motor learning [22].

In patients with dizziness, an important role is played by psychological factors. High levels of depression and anxiety were found in these patients with significant worsening of the quality of life and management of the pathology [11]. For this reason, psychological factors play a significant role in the recovery process in vestibular syndromes: some articles demonstrate the effectiveness of Cognitive Behavior Therapy (CBT) in directly or indirectly improving levels of anxiety and depression in specific populations with dizziness [23,24], but studies on how to improve treatment proposals are still ongoing [25].

In recent years, telerehabilitation (TR) has emerged as an innovative approach for delivering rehabilitation services remotely through communication technologies. Since the COVID-19 pandemic, the proportion of studies about this service [26] and its delivery showed an increase [27,28]. This growth is due to its potential in terms of accessibility, simplifying care delivery in unconventional healthcare frameworks [29,30] and, as in any other telemedicine service, cost and time savings [25]. TR services have been delivered in various areas of rehabilitation, such as musculoskeletal and neurological [31,32,33]. They encompass a wide range of activities, including assessment, monitoring, intervention, education, and more [34]. Telerehabilitation is defined as “the provision of rehabilitation services through information and communication technologies,” offering not only synchronous video conferencing but also asynchronous data sharing, enhancing its reach and effectiveness in rehabilitation [35]. In this review, both synchronous and asynchronous interventions delivered through various modalities (videoconferencing, use of platforms or websites, tele-support) were investigated. We choose to include tele-support among telerehabilitation interventions in line with the studies of Baroni et al. [31] and Cottrell et al. [35], who assert that the term “*Telerehabilitation*” is an overarching term encompassing all forms of remote rehabilitation that use information and communication technologies including tele-support. Telerehabilitation presents a series of characteristics that determine the modality of treatment delivery [34]. Specifically, it involves synchronous video conferencing, where telerehabilitation is delivered using audio-video technologies; asynchronous storage and sharing, where data on visits and diagnostic imaging are collected and shared; eConsult, which allows for the exchange of information on patient clinical questions through telecommunication; remote patient monitoring by healthcare personnel based on collected and transmitted data; and mobile health (*m*Health), which includes interventions delivered through mobile devices such as laptops or tablets, phone, or mobile phone.

While there is an increasing number of available studies on the evaluation [36,37] and management of dizziness through telemedicine [38,39], systematic reviews investigating the effectiveness of telerehabilitation in dizziness are still lacking, despite promising prospects [40,41] and the spread of TR services [27,28]. Therefore, this systematic review had two primary objectives: firstly, to provide an up-to-date overview of the topic, and secondly, to conduct a quantitative assessment of the effectiveness of telerehabilitation in treating dizziness. In this context, the primary outcomes under consideration were the frequency and severity of dizziness and disability, while the secondary outcomes focused on measures of anxiety and depression.

## 2. Materials and Methods

### 2.1. Protocol and Registration

The study design was set as a systematic review and meta-analysis and was conducted according to the Preferred Reporting Items for Systematic Reviews and Meta-Analyses 2020 (PRISMA) guidelines [42]. The protocol was registered a priori in the PROSPERO database under the following registration number: CRD42023451416.

### 2.2. Literature Search and Study Selection

The literature search was carried out up to 31 August 2023 in the following databases: MEDLINE (via PubMed), Cochrane Central Register of Controlled Trials, and PEDro. Specific search strategies for each database were employed (Supplementary Appendix SA). Furthermore, the references of relevant articles were also examined in order to find the greatest amount of useful and valid information.

Designed with the PICOs model [42], the structured questioning framework aimed at facilitating and strengthening search strategies [43]; the study focused on individuals experiencing dizziness (with or without other symptoms) as a clinical manifestation. The intervention investigated was telerehabilitation, and it was compared with conventional rehabilitation treatment as usual (TAU). Telerehabilitation was found to encompass various remote treatments, including remote rehabilitation sessions with a therapist, tele-consultation sessions with a therapist and individual treatment by the patient, alternating sessions of tele-consultation/rehabilitation and in-person sessions, monitoring through tele-consultation and in-person rehabilitative treatment, tele-consultation/rehabilitation sessions combined with other forms of treatment (face to face, internet-based interventions, digital platforms, or software), and asynchronous tele-consultation/rehabilitation sessions (internet-based). The primary outcome of interest in the study was the reduction in dizziness symptoms. Specifically, the primary outcomes focused on the reduction in both the frequency and severity of dizziness symptoms, measured using the Vertigo Symptom Scale—short form (VSS-SF) and the Vertigo Symptom Scale (VSS) [44,45]. The secondary outcomes included improvement of quality of life measured with the Dizziness Handicap Inventory (DHI) [46] and psychological impairments such as anxiety and depression measured with the Patient Health Questionnaire (PHQ) subscale [47], Hospital Anxiety and Depression Scale (HADS) [48,49,50], Generalized Anxiety Disorder assessment (GAD-7) [51] and Beck Depression Inventory Scale (BDI-II) [52,53]. More in detail, the Dizziness Handicap Inventory (DHI) and Vertigo Symptom Scale—short form (VSS-SF) are among the most commonly cited PRO instruments in clinical vestibular research [54], with the former one widely used even in the clinical context [41]. Whilst DHI explores each of the three ICF domains (activity, participation, body function and structures), VSS-SF focuses only on “body functions and structures” [54]. The remaining HADS, PHQ-9, GAD-7 and BDI-II have been frequently utilized in clinical vestibular research [24,55,56], considering the emotional, cognitive and psychological impacts of dizziness [57,58], which can result in a deterioration of the quality of life [59,60,61].

We included randomized controlled trials (RCTs) written in English or Italian. Excluded from our analysis were other study types, such as case reports or case studies, as well as studies involving children and adolescents (see Supplementary Appendix SB Table S1). The selection of studies was performed by two independent reviewers (DG)(MZ) according to the eligibility criteria (see Supplementary Appendix SB Table S1). The reviewers independently screened records that were identified, based on title and abstract, using an inclusion/exclusion criteria template. A third reviewer was selected to resolve any disagreements (SV). At the end of this process, the full text of the articles was obtained, and the same procedure was used for full text screening and for the assessment of the methodological quality of the studies.

### 2.3. Data Extraction

A data extraction form was filled with all the relevant data, i.e., authors and year of publication, number of participants and their characteristics (etiology, age, timing of symptoms), type of interventions and training, outcome measures assessed by authors (primary and secondary) according to our eligibility criteria (see Supplementary Appendix SB Table S1), effects of intervention and conclusions drawn by authors. Data were extracted independently by two authors (DG and MZ) and any divergences were resolved through a third author (SV). Furthermore, the studies were divided into synchronous and asynchronous telerehabilitation.

### 2.4. Quality Assessment

The included studies were assessed for their quality using the Revised Cochrane risk of bias tool for Randomized Trials (RoB2) [62] by two authors (DG and BC). Five domains were assessed: (a) selection bias, (b) performance bias, (c) detection bias, (d) attrition bias, and (e) reporting bias. For each domain, the risk of bias was coded into one of the three following possibilities: low, low risk of bias; high, high risk of bias; some concerns, when the reporting was insufficient and some concerns were raised. Finally, potential publication bias was explored through visual inspection of funnel plots.

### 2.5. Data Analysis

Statistical analysis and meta-analysis calculations were carried by two authors (MZ and BC) by utilizing RevMan 5.4, the Cochrane software review manager for writing and carrying reviews (currently available via subscription) [63]. In the conducted meta-analysis, attempts were made to categorize the interventions into four outcome groups: frequency and severity of dizziness assessed with VSS-SF [44,45] (primary outcome); improvement of quality of life measured with DHI [46] (secondary outcome); psychological impairments (secondary outcomes) categorized into anxiety measured with HADS-A [48,49,50], BDI-II [52,53], and GAD-7 [51,64]; and depression measured with HADS-D [48,49,50] and PHQ-9 [47]. The data point for the processing outcome meta-analysis data was considered to be three months. Mean difference (MD) outcome measures were used for the analysis where the study used the same tools. Standard mean difference (SMD) outcome measures were used for the analysis since the selected studies used different tools. When possible and when not reported, the MD and standard deviation (SD) were estimated from the standard error (SE) of the mean or median and interquartile range. Forest plot graphics were generated to demonstrate the pooled effect. Heterogeneity was assessed using the I^2^ statistic and was categorized as low if I^2^ < 25%, moderate if I^2^ was between 25 and 50%, and high if I^2^ > 50% [65]. In the case of no data being available for synthesis, an email was sent to the corresponding author. We assumed a 2-week waiting period for a response. We planned a subgroup analysis in relation to synchronous and asynchronous treatment. In the depression outcome group, it was not possible to perform subgroup analysis. We used a fixed-effects model in our meta-analysis because we assumed that all included studies were estimating the same underlying true effect size. This approach is appropriate when there is minimal heterogeneity among studies, and we aimed to provide a precise estimate of the common effect size [65]. In cases where significant heterogeneity exists among studies, we opted for a random-effects model. This model accounts for both within-study and between-study variability, allowing for a more conservative estimate of the overall effect size that can accommodate differences in study populations, methodologies, or other factors contributing to heterogeneity.

## 3. Results

### 3.1. Article Selection Process

**Figure 1:**
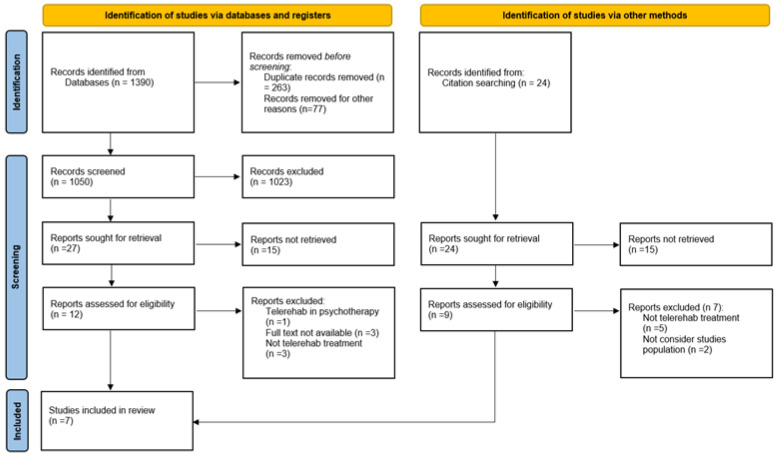
Flow diagram for the study selection process.

The search strategy yielded a total of 1414 articles, including 331 from PubMed, 356 from the Cochrane Central Register of Controlled Trials, and 703 from PEDro. Additionally, 24 records were identified through cross-reference sources. Of the 1390 articles initially identified in the databases, 263 were removed due to duplication, and 77 were excluded as they were in a different language, leaving 1050 articles for screening. Of these, 1023 were excluded because they were either not relevant to the topic of interest or had an inadequate study design, leaving 27 abstracts for further evaluation. After reviewing these abstracts, 14 articles were excluded as they did not pertain to the clinical question. Subsequent full-text review led to the exclusion of eight more articles. Regarding the 24 articles identified through other methods, such as cross-referencing, 15 were excluded after abstract review due to irrelevance or inadequate study design. Out of the nine remaining, seven were further excluded after full-text review. Ultimately, at the end of the selection process, seven eligible articles remained: five from the database screening and two identified through other methods. The selection process is detailed in the PRISMA 2020 flowchart [42], presented in Figure 1. The two authors demonstrated a remarkable level of consensus (K = 0.93, 95% CI 0.80–1.00). At the end of the full text reading stage, a disagreement on one study [66] initially included was solved, and it was finally excluded because of its unsuitable study design. The list of excluded studies is provided in the Supplementary Section (Supplementary Appendix SB Table S2). This compilation, represented in Table S2, encompasses 15 studies that were initially considered for inclusion but were subsequently excluded after a comprehensive full-text examination.

### 3.2. Results of Selected Articles

The seven included studies were published between 2004 and 2023 and are summarized in the table below (Table 1). All articles are RCTs, three of which are pragmatic [67,68,69], investigating different modes of telerehabilitation, both as an intervention (synchronous or asynchronous) and as tele-consultation and/or monitoring, proposed as a single intervention or combined with other interventions. In the studies, the intervention is compared to usual care, mainly in the pragmatic trials (reassurance plus medication for symptom reduction), or to an exercise program. The study population includes patients with BPPV [70], stable vestibular disorders, both peripheral and central or mixed [71,72] and chronic vestibular disorders/vertigo [67,68,69,73].

Four of the included studies used asynchronous telerehabilitation, through the use of websites that deliver the intervention without requiring real-time presence of the therapist: Van Vugt et al. [68] and Geratghy et al. [67] used the “Balance Retraining” program in their pragmatic RCTs, while Smaerup et al. [71,72] used the exercise platform “Move it to improve it” after installing the corresponding hardware and software at the patient’s home. The “Balance retraining” program is based on the content of a booklet used in previous trials [69,73] downloadable from the Supplementary Data of the original article or from the dedicated page of the Meniere’s Society website. The program includes the administration of exercises of different difficulty, from cervical spine mobility to gaze stability with open and closed eyes to exercises related to provocative daily movements. Sessions on symptom control techniques are provided. Based on the scores of various performances, the online program will increase the difficulty level of subsequent sessions [74]. The Mitii program involves playing games (drag and drop and follow the leader) with the goal of training endurance, VOR, and cervico-ocular reflex (COR) for gaze stability, smooth-pursuit eye movements, and postural control. The therapist contacts the patient once a month to adjust the exercise variables based on progress [75].

Geraghty et al. [67], in a population with chronic vestibular disorders, compares the online vestibular rehabilitation program to usual care. In his study, the intervention group showed improvement in VSS-SF values compared to usual care at 3 (*p* < 0.001) and 6 months (*p* = 0.02). Van Vugt et al. [68], using three distinct arms, compares online vestibular rehabilitation with or without physiotherapy support to usual care. In this study, when compared to usual care at 3 months, the group that only performed telerehabilitation showed a greater difference in VSS-SF (Intention to Treat analysis −4.3 points, CI −5.9; −2.6) as well as the group that performed telerehabilitation and had two in-person sessions with the physiotherapist (ITT −3.9 points, CI−5.5; −2.3). Smaerup et al. [71,72] compare their online rehabilitation program to a paper-based program delivered to patients for independent sessions, in a total population with stable vestibular disorders (peripheral, central, or mixed) who are already receiving face-to-face rehabilitation in clinic twice a week. At the end of this, the authors set up another trial [72] to compare any changes at 3 months between those who continued with online rehabilitation and those following the paper instructions.

In a population of patients with stable vestibular disorders, its application did not show any statistically significant differences in DHI (*p* = 0.212) and other proposed balance tests, i.e., one-leg stand test (*p* = 0.755), compared to independently performing exercises through a paper program [71]. This difference was not achieved during a 16-week period in which both groups underwent two additional in-person therapy sessions, or in the following 12 weeks (during which the patients continued with the same exercise program) [72]. Haciabbasoğlu et al. [70] investigate the effectiveness of balance and adaptation exercises performed through synchronous telerehabilitation, plus independently performed vestibular rehabilitation adaptation exercises, compared to only vestibular rehabilitation adaptation exercises performed at home. The intervention group’s program includes exercises for gaze stability, imagery pursuit eye movement, and static and dynamic balance (Romberg, Tandem, Semi-tandem, walking), delivered by the therapist through WhatsApp video calls and also performed independently without therapist support. Intervention group participants show a statistically significant difference from control in Tandem closed eyes (*p* = 0.022) and DHI (*p* < 0.0001).

Yardley et al. investigate, in two studies [69,73], the effectiveness of a self-managed exercise program based on a booklet, with or without tele-support sessions (respectively, two and three sessions in the two trials), compared to usual care, in a population of patients with chronic vertigo. The booklet contains information about one’s condition, instructions for performing exercises, symptom management and progression, and a schedule/diary for planning weekly exercise sessions. In a 2012 study, Yardley et al. [69] use three distinct arms: “Book self-management and telephone support”, “Book self-management”, and “Routine care. The study shows that an exercise program based on a booklet, with telephone support, shows a greater reduction in VSS-SF (*p* = 0.014), HANDS depression (*p* = 0.016), and HANDS anxiety (*p* = 0.014) at one year compared to usual care, while no statistically significant difference between “Booklet self-management with telephone support” and “Routine care” at VSS-SF, HANDS depression and HANDS anxiety at 12 weeks is reported. The “Book self-management and telephone support” group had the best cost-effectiveness curve in the author’s analysis. In 2004, Yardley et al. [73] compared two groups, one with “Book self-management and telephone support” and one with “Usual medical care group”. The group that performed “Book self-management and telephone support” showed a greater difference in VSS-SF score at 3 (*p* < 0.001) and 6 months (*p* = 0.004) compared to the other group.

### 3.3. Methodological Evaluation of Studies

**Figure 2:**
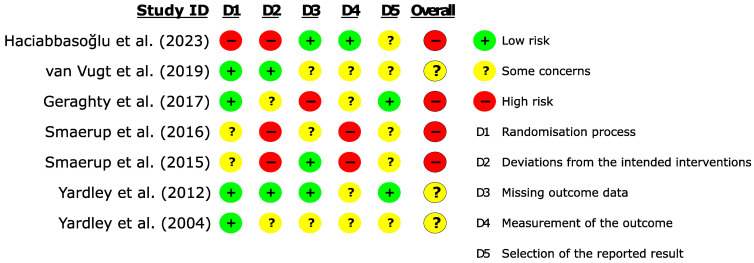
Risk of bias in the included studies.

Figure 2 shows the risk of bias in the included studies. Of the seven included articles, the overall risk of bias was found to be moderate for three articles [68,69,73] and high for the remaining four articles [67,70,71,72]. The “risk of bias in the randomization process” was low in four articles [67,68,69,73]. It was moderate in two articles [71,72] where no significant differences were noted between the two groups despite the absence of information regarding adequate allocation sequence masking. It was high in one article [70] where significant differences in initial characteristics between the two groups were present. The risk of bias due to deviations from intended interventions was low in two studies [68,69] because the interventions delivered were consistent with the planned and protocol-reported interventions. Geraghty et al. [67] employed “Intention to Treat” analyses (considered an appropriate analysis tool to assess intervention effects), but no additional information was available for its management, resulting in a moderate risk in this domain. The remaining three studies declared themselves as single-blind: only Haciabbassoglu et al. [70] used participant blinding (although no strategy was explained), while two studies by Smaerup et al. [71,72] used assessor blinding. Therefore, they were considered to be at high risk. The risk of bias due to missing data was low in three studies [47,48,49]. Some concern arose in three studies [68,72,73] where authors decided to alter the analysis strategies outlined in the protocol in response to missing data. High risk was noted in one study [67] where a significant dropout imbalance in favor of the intervention group occurred at 3 and 6 months, without a clear explanation from the author for the lost participants. Outcome measurement bias was low risk in one study [70] where the outcome assessor was blinded (i.e., the patient). It was moderate risk in four studies [67,68,69,73] where the outcome assessor was not blinded. It was considered high risk in the two RCTs of Smaerup et al. [71,72] where no useful information was reported to determine the psychometric extent to which the assessor’s knowledge of the intervention influenced the final outcome. Outcome reporting bias was at low risk in two studies [67,69]. In van Vugt et al. [68], it had a moderate risk due to the choice of using a different type of statistical analysis for managing missing data than what was specified in the protocol. The same moderate risk was present in three articles where the type of statistical analysis was not described in advance in the study protocol [70,71,72], and one article [73] where the protocol was missing entirely.

### 3.4. Effects of Intervention

#### 3.4.1. Frequency and Severity of Dizziness

**Figure 3:**
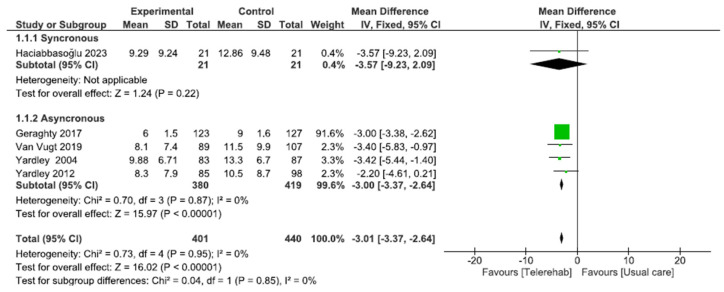
Telerehabilitation versus usual care treatment for frequency and severity of dizziness. A green block indicates the weight assigned to the study, and the horizontal line depicts the confidence interval. Black rhombi show the overall results.

We included five studies with a total of 841 participants. Given the use of same assessment tool, VSS-SF, we used mean difference with a fixed effect model. A subgroup analysis was performed by dividing the five studies into synchronous (one study) and asynchronous (four studies). The total result showed a significant difference in favor of the telerehabilitation group (MD of −3.01; CI −3.37; −2.64; I^2^ = 0%, *p* < 0.001) as well as for all the subgroups, i.e., synchronous (MD of −3.57; CI −9.23; −2.09; *p* = 0.22) and asynchronous (MD of −3.01; CI −3.37; −2.64; I^2^ = 0%; *p* < 0.001) (Figure 3).

#### 3.4.2. Disability

**Figure 4:**
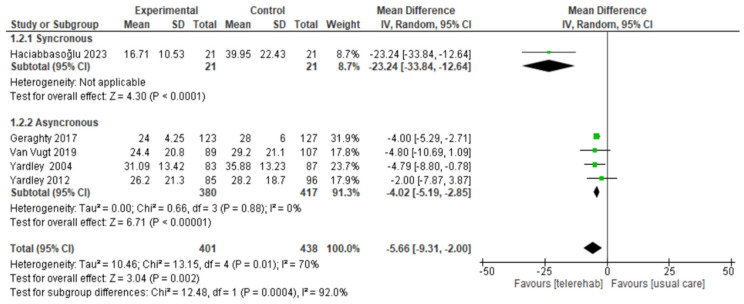
Telerehabilitation versus usual care treatment for disability treatment. A green block indicates the weight assigned to the study, and the horizontal line depicts the confidence interval. Black rhombi show the overall results.

We included five studies with a total of 839 participants. Given the use of the same assessment tool, DHI, we used mean difference with a random effect model. A subgroup analysis was performed by dividing the five studies into synchronous (one study) and asynchronous (four studies). The total result showed a significant difference in favor of the telerehabilitation group (MD of −4.25; CI −5.42; −3.09; I^2^ = 70%, *p* < 0.001) as well as for all the subgroups, i.e., synchronous (MD of −23.24; CI −33.84; −12.64; *p* = 0.0001) and asynchronous (MD of −4.02; CI −5.19; −2.85; I^2^ = 0%; *p* < 0.001) (Figure 4).

#### 3.4.3. Anxiety

**Figure 5:**
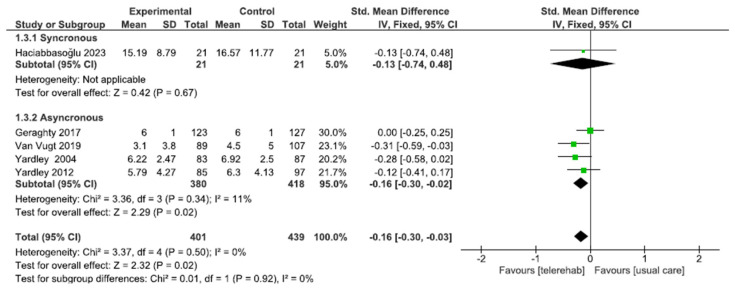
Telerehabilitation versus usual care treatment for anxiety treatment. A green block indicates the weight assigned to the study, and the horizontal line depicts the confidence interval. Black rhombi show the overall results.

We included 5 studies with a total of 840 participants. Due to the different anxiety assessment tools used in the included studies: HADS anxiety, BAI, and GAD-7, the analysis was performed using SMD with a fixed effect model. A subgroup analysis was performed by dividing the five studies into synchronous (one study) and asynchronous (four studies). The total result showed a significant difference in favor of the telerehabilitation group (SMD of −0.16; CI −0.30; −0.03; I^2^ = 0%, *p* = 0.02). In the subgroup analysis, synchronous treatment shows no significant difference with respect to usual care (SMD of −0.13; CI −0.74; −0.48; *p* = 0.42) while asynchronous treatment shows a significant different with respect to usual care (SMD of −0.16; CI −0.30; −0.02; I^2^ = 11%; *p* = 0.02) (Figure 5).

#### 3.4.4. Depression

**Figure 6:**
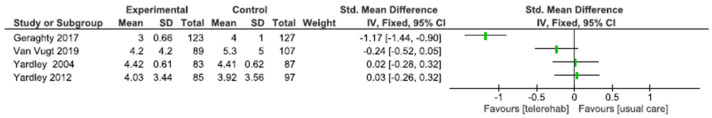
Telerehabilitation versus usual care treatment for depression treatment. A green block indicates the weight assigned to the study, and the horizontal line depicts the confidence interval. Black rhombi show the overall results.

We included 4 studies with a total of 798 participants. Due to the different depression assessment tools, HADS depression and PHQ-9, used in the included studies, the analysis was performed using SMD with a fixed effect model. The result showed a non-significant difference between the treatment groups (Figure 6). Only one study [67] showed to be more effective than the control group. Due to the high heterogeneity of the data, it was impossible to assess the pulled results.

## 4. Discussion

Considering the recent advancements in telerehabilitation across various fields, this review aimed to investigate the efficacy of telerehabilitation in treating dizziness. The results from the review indicate evidence supporting its effectiveness in reducing the frequency and severity of dizziness, as well as associated disability and anxiety levels. To the best of our knowledge, this is the first review conducted on this specific topic. While there are similar reviews in the literature, none have examined the use of telerehabilitation in the specific population of patients with dizziness. Beukes et al. [76] focused solely on internet-based interventions for a broader population, including adults with hearing loss, tinnitus, and vestibular disorders. Two other reviews [77,78] did not include telerehabilitation among their interventions, and two others [79,80] examined different populations. Additionally, the review by Gaikwad et al. [81] did not investigate the efficacy of telerehabilitation for dizziness but rather focused on the best adherence strategies for home exercises.

This review of the articles identified two telerehabilitation treatment modalities: synchronous and asynchronous. In developing the meta-analysis, we decided to include the two studies of Yardley et al. [69,73] among the interventions performed asynchronously. The results of the meta-analysis highlight, for the primary outcome: frequency and severity of dizziness (measured with VVS-SF), a statistically significant difference in the tele-rehabilitation group compared to the control group. The secondary outcomes considered were disability, levels of anxiety and depression. Concerning the disability outcome, measuring with DHI also showed a statistically significant difference in the telerehabilitation group compared to the control group, but this result must be considered in light of the high heterogeneity found for anxiety outcomes assessed with different tools: HADS anxiety in three studies [67,69,73] and BDI [70] and GAD-7 [68] in one study each. The studies showed a statistically significant difference in the telerehabilitation group compared to the control group. Depression outcomes were assessed with different methods: HADS depression in three studies [67,69,73] and PHQ-9 in one study [68]. This showed a non-statistically significant difference in the telerehabilitation group compared to the control group, as indicated by the meta-analysis; however, this result must also be considered in light of the high heterogeneity found. From the subgroup analyses, it was seen that the asynchronous mode was considered more effective in reducing the frequency and severity of dizziness and anxiety compared to the synchronous modality. Furthermore, these results need to be considered carefully due to the presence of only study [70] in synchronous compared to four in asynchronous [67,68,69,73].

In addition, all the results should be interpreted in light of the medium-low methodological quality of the studies. Four studies [67,70,71,72] had a high overall risk of bias due to serious doubts arising, respectively: randomization and deviation from the planned intervention in Haciabbasoglu et al. [70], data management in Geraghty et al. [67], and deviation from the planned intervention and outcome measurement in the two Smaerup et al. trials [71,72]. Three studies [68,69,73] had a medium risk of bias due to concerns regarding data management, outcome measurement, and selection of reported results in Van Vugt [68]; outcome measurement in Yardley et al.’s 2012 study [69]; and deviation from the planned intervention, data management, outcome measurement, and selection of results in Yardley et al.’s (2004) study [73]. This could explain high heterogeneity in the meta-analysis that can be found in disability and depression outcomes.

The proposed interventions analyzed in the studies have various differences among them. Firstly, the modes of telerehabilitation usage vary, with four studies [67,68,71,72] using asynchronous mode, one study using synchronous mode [70] and two studies [69,73] using tele-support. Among the asynchronous studies, out of the two studies [67,68] that use the balance retraining program, only Van Vugt et al. [68] provides more detailed information on the dosage (6 weeks of daily sessions with 6 online VR exercises for 10 min, twice a day, plus different weekly sessions of online VR). Geraghty et al. [67], on the other hand, only report 6 weeks of online VR provided by the website. On the other hand, the type of intervention delivered by the online program is similar not only between the two mentioned studies but also to the intervention proposal in Yardley’s two trials [69,73], as the online program is based on the validated booklet from Yardley et al.’s (2004) study [73]. The exercise dosage proposed by Yardley et al. (2012) [69] based on the booklet is considered similar to that of Van Vugt et al. [68] (daily VR exercise sessions of 5–10 min in Yardley et al. (2012) [47] and 10 min in Van Vugt et al. [68], both for 6 weeks), although the two programs differ in the addition of different exercises.

Regarding the usability and acceptance of these tools, qualitative studies in the literature have investigated the perspective of physiotherapists engaged in vestibular physiotherapy through telerehabilitation platforms. Harrell et al. [40] collected the experiences of 159 therapists in the United States who treated central or peripheral vestibular syndromes using online questionnaires. Eighty-six percent of respondents (“strongly agree” and “somewhat agree”) considered “telehealth” an effective means of delivering vestibular physiotherapy, 56% (“strongly agree” and “somewhat agree”) believed they had a similar participation compared to in-person rehabilitation, and 68% (“strongly agree” and “somewhat agree”) reported achieving similar results to face-to-face sessions.

Another survey by Meldrum et al. [82], including responses from 471 physiotherapists from 20 different European countries, indicated general difficulty in accessing knowledge and resources for vestibular rehabilitation. Only 4.5% reported using telerehabilitation in this context. A study by Muller et al. [83] explored the experiences of patients undergoing vestibular rehabilitation with telephone support in Yardley et al.’s research [69,73]. Through a questionnaire, 33 patients were asked about “living with dizziness, the experience of rehabilitation, and barriers and outcomes of treatment.” In the “treatment experience” section, the impact of telephone support on these patients was investigated. A large proportion reported that they would have benefited from additional telephone support, as it made them feel more motivated and adherent to exercise and helped build a therapeutic relationship with their therapist. To date, further platforms for the rehabilitation of vestibular disorders are emerging: information on tools still in the initial stage of experimentation can be retrieved from gray literature, such as WeBaVer and RehaMetrics^®^, a [83] Norwegian platform created by Molde Hospital [84] and VestAid [85].

### 4.1. Study Limitations

This literature review has several limitations. Firstly, there is a certain heterogeneity in the type of intervention, both in terms of telerehabilitation methods and types and dosage of vestibular rehabilitation exercises. On the one hand, this makes it difficult to generalize the effectiveness of telerehabilitation in vestibular syndromes due to the lack of consistency in the interventions performed. On the other hand, this variety of programs could potentially represent an opportunity for personalized proposals based on the clinical characteristics of our patient and context. However, it is important to note that while some of these treatments have shown effectiveness in reducing dizziness symptoms in certain population groups (such as BPPV or chronic vestibular disorders), it is not possible to establish whether they would be equally effective with other population groups not examined in this study (e.g., cervicogenic vertigo). Lastly, the medium-low methodological quality of the included studies affects the possibility of transferring and generalizing these treatment modalities in a clinical practice setting.

### 4.2. Clinical Implication and Future Study Directions

Dizziness is a highly debilitating symptom. Individuals who suffer from it may experience a reduction in quality of life [59,60,61] and sleep, cognitive impairments, fear of movement, and increased risk of developing anxiety, depression, and panic attacks [57,58,59,86]. Physiotherapeutic intervention is safe, effective, and free from serious adverse effects, reduces the need for medication, and reduces the occurrence of dizziness in the medium to long term [21]. Therefore, it is recommended for various types of dizziness [87]. Similarly, a rehabilitation intervention performed through telerehabilitation could have the same clinical benefits with the addition of increased long-term patient compliance and remote patient monitoring by a physiotherapist. The value of the analysis conducted is particularly evident in the era of the COVID-19 pandemic, where telerehabilitation has been seen to have great importance. Nevertheless, in normal situations, it could increase the patient’s compliance with long-term treatment, thus helping them reduce the symptoms that, in these pathologies, are often recurring. Furthermore, it could be useful for those people who cannot reach the clinics and live in hard-to-reach places. It would also allow the clinic to monitor the patient over time and modulate the treatment according to its needs. Taking into account the study limitations, clinicians could benefit from our work, since a variety of effective rehabilitation program and platform references, suitable for TR purpose, are reported.

For future clinical studies, it is recommended to investigate the effects of telerehabilitation on a population with dizziness with greater methodological rigor. Any new studies should analyze the different modes of telerehabilitation (synchronous, asynchronous, remote support, and monitoring), for each of which it would be necessary to standardize the use of platforms, exercise programs, and related dosages as much as possible. Considering the varied etiology of the condition under examination, it might be useful to evaluate the treatment effects on different subgroups of the population to detect any differences in terms of effectiveness.

## 5. Conclusions

The results of this review regarding the effect of telerehabilitation in dizziness are potentially in line with what has already been observed in other studies [17,88,89] on vestibular rehabilitation in the presence of BPPV or chronic vestibular disorders. In fact, this digital delivery method is shown to be effective in treating dizziness in a population of patients with BPPV, particularly with a synchronous model and self-adaptation exercises. Asynchronous models [67,68,71,72] or telephone support [69,73] are also effective in reducing dizziness symptoms in a population with chronic vestibular disorders. Due to the medium-low methodological quality of the included articles and the heterogeneity of telerehabilitation methods, dosages, and exercise programs within them, further studies will be necessary to define the real effectiveness of the individual modes using standardized platforms and programs for different patient populations characterized by these clinical manifestations.

## Figures and Tables

**Table 1:** Characteristics of included studies.

Author and Year	Population	Interventions	Outcome Measures	Effects (Experimental vs. Control Comparison)	Conclusion
Haciabbasoğlu et al., 2023 [70]	44 individuals with diagnosis of “positional vertigo”. Age = 18–65 years. Experimental group: 22 individuals. Control group: 22 individuals.	Experimental group: Vestibular rehabilitation adaptation exercises performed at home, and vestibular rehabilitation balance exercises in telerehabilitation. Telerehabilitation with therapist via WhatsApp video call, 25–30 min 2 times a day for 6 weeks. Exercises in frequency autonomy not specified. Control group: Vestibular rehabilitation adaptation exercises performed at home 2–3 times/day for 6 weeks. The exercises were shown in person, and sent via WhatsApp together with other material additional	Primary Outcome: - Romberg test. - Tandem posture test (open and closed eyes). - Semi Tandem Posture Test (open and closed eyes). - DHI. - Dizziness rating. - VSS-SF. - BAI. - VDI.	Primary Outcome (6 weeks): Romberg: *p* = 0.593 (z = −0.535) Tandem Open: *p* = 0.757 (z = −0.309) Tandem Closed: *p* = 0.022 (z = 2.287) Semi Tandem open: *p* = 0.973 (z = −0.034) Semi Tandem closed: *p* = 0.054(z = −1.928) DHI: *p* = 0.0001(t = 4.298) VSS-SF: *p* = 0.06 (z = −1.878) BAI: *p* = 0.669 (t = 0.431) VDI: *p* = 0.004 (t = 3.071)	TR applications are effective and clinically applicable in patients with BPPV.
Van Vugt et al., 2019 [68]	322 individuals aged visited by the GP in the previous 2 years for vestibular symptoms. Age > 50 years. Experimental group: 98 individuals. Control group: 120 individuals.	Experimental group: “Stand alone internet-based VR” 6 weeks of: daily sessions of 6 online VR exercises independently provided by the “Balance Retraining” site (10 min × 2 times a day) without support from the therapist. Different weekly sessions of online VR without therapist support. Information and advice on anxiety control strategies. Weekly email to remind you to access the site. Control group: “Usual care”: standard level of care provided by their doctor, with access to every available treatment between primary and secondary care after referral.	Primary Outcome: - VSS-SF. Secondary Outcomes: - DHI. - SINGLE ITEM on perception of being improved or not. - PHQ. - (GAD-7) subscales. - (PHQ-9 subscale). - PETS (only on intervention).	Primary Outcome (3 months): Stand-Alone VR vs. usual care VSS -SF: −4.3 points (95% CI −5.9 to −2.6) Blended VR vs. Usual care VSS-SF: −3.9 points (95% CI −5.5 to −2.3) Secondary Outcome (3 months) Stand-Alone VR vs. usual care DHI: −4.6 points (95% CI −8.2 to −1.1) SINGLE ITEM: 2.2% (95% CI 1.2 to 4.1) PHQ-9: −0.5 points (95% CI −1.4 to 0.4) GAD-7: −1.1 points(95% CI−1.9 to −0.3) Blended VR vs. usual care DHI: −3.9 points (95% CI −7.4 to −0.4) SINGLE ITEM: 2.1% (95% CI 1.2 to 3.8) PHQ-9: −0.9 points (95% CI −1.8 to 0.0) GAD-7: −1.4 points(95% CI−2.2 to −0.6)	Stand-alone and blended internet-based VR are clinically effective and safe interventions to treat adults aged 50 and older with a chronic vestibular syndrome.
Geraghty et al., 2017 [67]	296 individuals visited the GP for vertigo in the last 2 years, and with still present vertigo which worsens with movement of the head. Age > 50 years. Experimental group:160 individuals. Control group: 136 individuals.	Experimental group: 6 weeks of online VR provided by the site “Balance Retraining” without therapist support.—information and advice on anxiety control strategies. Control group: “Usual Care”: subjects receive usual UK primary care, i.e., reassurance, symptom relief (e.g., medication for nausea) and sometimes education.	Primary Outcome: - VSS-SF. Secondary Outcomes: - VSS-SF vertigo sub scale score - VSS-SF autonomic sub scale score. - DHI. - HADS anxiety score. - HADS depression score. - SINGLE ITEM on perception of being improved or not.	Primary Outcome (3 months): VSS-SF total: −2.75 points (95% CI −1.39 to −4.12, *p* < 0.001) Secondary Outcome (3 months) VSS-SF vertigo subscale: −1.49 points (95% CI −0.54 to −2.43; *p* = 0.002) VSS-SF autonomic subscale: −1.03 points (95% CI – 0.12 to −1.94; *p* = 0.03) DHI: −6.15 points (95% CI −2.81 to −9.49; *p* < 0.001) HADS anxiety: −0.82 points (95% CI −0.03 to −1.61; *p* = 0.04) HADS depression: −0.55 points (95% CI 0.18 to −1.28; *p* = 0.18) SINGLE ITEM: 0.27% (95% CI 0.17 to 0.44; *p* < 0.001)	Internet-based vestibular rehabilitation reduces dizziness and dizziness-related disability in older primary care patients without requiring clinical support.
Smaerup et al., 2016 [72]	63 individuals who completed the inpatient rehabilitation program. Individuals with peripheral, central or mixed stable vestibular disorder. ≥65 years. Experimental group: 32 individuals Control group: 31 individuals	Experimental group: Intervention provided by the “Mitii” website through a PC connected to the Internet and a webcam. Daily exercise program of 20/30′, at least once a day, with sequences of games. The site sends information on the duration of the treatment to the hospital therapist, who contacts the patient in the event of a 7-day absence from the program. The therapist prompts participants to continue with exercise sessions even after they finish. Twelve-week follow-up. Control group: Standard care: after hospital discharge, printed instructions are given for continuing the exercises at home. Exercise program of 20/30′. Twelve-week follow-up.	Primary outcome: - One leg stand test. Secondary outcome: - Dynamic Gait Index. - DHI. - Short Form 12. - Motion Sensitivity test. - VAS. - Chair Stand Test.	Primary outcome (12 weeks): One leg stand test: −1.26 s (95% CI −4.07 to 1.56, *p* = 0.38) Secondary outcome (12 weeks): Dynamic Gait Index: −0.35 *p* (95% CI −1.48 to 0.78, *p* = 0.54) DHI: −0.67 points (95% CI −6.43 to 5.07, *p* = 0.81) SF-12: −1.46 points (95% CI −4.07 to 1.16, *p* = 0.58) Motion Sensitivity test: −0.26 points (95%CI −4.20 to 3.68, *p* = 0.12) VAS = 0.53 mm (95%CI −9.51 to 10.56, *p* = 0.92) Chair Stand Test: 0.50 rep (95% CI −0.71 to 1.72, *p* = 0.41)	Elderly vestibular dysfunction patients exercising at home seem to maintain their functional level, level of dizziness, and quality of life three months following discharge from hospital. In this specific setup, no greater effect was found by introducing a computer-assisted training program, when compared to standard home training guided by printed instruction.
Smaerup et al., 2015 [71]	63 individuals underwent in-hospital rehabilitation 2 times a week for 16 weeks, with diagnosis peripheral, central or mixed stable vestibular disorder. Age ≥ 65 years. Experimental group: 32 individuals. Control gropu:31 individuals.	Experimental group: Intervention provided by the “Mitii” website through a PC connected to the Internet and a webcam. Daily exercise program of 20/30′ with sequences of games for 16 weeks. The site sends information on the duration of the treatment to the hospital therapist, who contacts the patient in the event of a 7-day absence from the program. The therapist calls once a month to adjust the duration, speed, and difficulty of the exercises based on progress. The patient is also undergoing rehabilitation in hospital 2 times/week for 16 weeks. Control group: Delivered a paper program of the exercises to be performed, of 20/30′ at least once a day, for 16 weeks.	Primary Outcome: - One leg stand test. - Dynamic Gait Index. - DHI. - SF 12 physical functioning. - SF 12 mental functioning. - Motion Sensitivity test - VAS - Chair Stand Test.	Primary outcome (16 weeks): One leg stand test: −0.55 s (95% CI −4.06 to 2.96, *p* = 0.755) Secondary outcome (16 weeks): Dynamic Gait Index: −0.17 *p* (95% CI −1.74 to 1.41, *p* = 0.833) DHI: −4.73 points (95% CI −12.23 to 2.77, *p* = 0.212) SF-12 pf: −0.48 points (95% CI −5.60 to 4.64, *p* = 0.851) SF-12 mf: −2.24 points (95% CI −3.16 to 7.64, *p* = 0.410) Motion Sensitivity test: −3.33 points (95%CI −13.71 to 7.04, *p* = 0.523) VAS = 0.37 mm (95%CI −11.08 to 11.82, *p* = 0.949) Chair Stand Test: 0.00 rep (95% CI −1.31 to 1.31, *p* = 1.000)	A computer-assisted program to support the home training of elderly patients with vestibular dysfunction did not improve rehabilitation more than printed instructions did.
Yardley et al., 2012 [69]	337 individuals with dizziness in the last 2 years. Age ≥ 18 years. Experimental group 112 individuals. Experimental group: 113 individuals. Control group: 112 individuals.	Experimental group: “Book self-management and telephone support” group, with exercise sessions conducted through a validated booklet, of 5–10′ twice a day for 12 weeks, plus 3 telephone support sessions at baseline, first and third week. Follow up at 12 weeks and 1 year. Experimental group: “Book self-management” group with exercise sessions carried out through a validated booklet, of 5–10′ twice a day for 12 weeks. Follow up at 12 weeks and 1 year. Control group: “Routine care” group, treated with reassurance and symptom reduction (e.g., drugs). Follow up at 12 weeks and at 1 year.	Primary outcome: - VSS-SF. - Cost/utility through cost per QALY. Secondary outcome - Subjective improvement of vertigo. - Vertigo balance subscale - Autonomic Anxiety and Depression scale - DHI. - HADS - EuroQol-EQ-5D - Problematic experiences of therapy scale.	Primary outcome (12 weeks): Book self-management and telephone support vs. routine care VSS-SF: −1.79 points (95% CI −3.69 tο 0.11, *p* = 0.064) Booklet self management only vs. routine care VSS-SF: −0.59 points (95% CI −2.45 tο 1.27, *p* = 0.532) Secondary Outcome (12 weeks): Book self-management and telephone support vs. routine care Subjective improvement: 2.25 OR (95% CI 1.28 to 3.94, *p* = 0.005) Vertigo balance subscale: −0.74 points (95% CI −1.98 tο 0.51, *p* = 0.246) Autonomic Anxiety subscale: −1.11 points (95% CI −2.03 tο −0.20, *p* = 0.017) DHI: −2.25 points (95% CI −5.98 tο 1.47, *p* = 0.234) HADS (anxiety): −0.46 points (95% CI −1.21 tο 0.29, *p* = 0.228) HADS (depression): −0.02 points (95% CI −0.66 tο 0.62, *p* = 0.954) EQ-5D: 0.04 points (95% CI −0.02 tο 0.10, *p* = 0.156) Booklet self-management only vs. routine care Subjective improvement: 2.41 OR (95% CI 1.39 to 4.20, *p* = 0.002) Vertigo balance subscale: −0.46 points (95% CI−1.67 tο 0.75, *p* = 0.454) Autonomic Anxiety subscale: −0.08 points (95% CI −1.00 tο −0.82, *p* = 0.869) DHI: −2.06 points (95% CI −5.74 tο 1.61, *p* = 0.269) HADS (anxiety): −0.12 points (95% CI −0.88 tο 0.65, *p* = 0.763) HADS (depression): −0.28 points (95% CI −0.93 tο 0.37, *p* = 0.396) EQ-5D: 0.04 points (95% CI −0.02 tο 0.09, *p* = 0.179)	Booklet-based vestibular rehabilitation for chronic dizziness is a simple and cost-effective means of improving patient-reported outcomes in primary care.
Yardley et al., 2004 [73]	170 individuals with vertigo in the last two years. Age ≥ 60 years. Experimental group: 83 individuals. Control group: 87 individuals.	Experimental group: “Vestibular rehabilitation group” with exercise sessions carried out through a booklet, plus two telephone support sessions in the first and third week. Follow up at 12 weeks and 6 months. Control group: “Usual medical care group” treated with reassurance and symptom reduction (e.g., drugs). Follow up at 12 weeks and at 6 months.	Primary Outcome: - VSS-sf - Movement provoked dizziness. - Postural Stability, eyes open. - Postural stability, eyes closed. - DHI. Secondary outcomes: - SF 36 physical functioning. - HADS.	Primary Outcome (3 months): VSS-SF: −3.48 points (95CI −5.59 to −1.38, *p* = 0.001) Movement provoked dizziness: −6.15 points (95% CI −9.40 to −2.90, *p* = 0.001) Postural stability (open): −65.00 mm (95% CI −119.01 to −11.00, *p* = 0.019) Postural stability (closed): −122.29 mm (95% CI −209.85 to −34.74, *p* = 0.006) DHI: −4.78 points (95% CI −8.98 to −0.59, *p* = 0.026) Secondary Outcome (3 months): SF-36: 1.18 points (95% CI −0.09 to 2.46, *p* = 0.069) HADS (anxiety): −0.70 points (95% CI −1.48 to 0.08, *p* = 0.079) HADS (depression): 0.01 points (95% CI −0.19 to 0.21, *p* > 0.2)	Booklet based vestibular rehabilitation for chronic dizziness is a simple and cost-effective means of improving patient reported outcomes in primary care.

BPPV: Benign Paroxysmal Positional Vertigo, DHI: Dizziness Handicap Inventory, VSS-SF: Vertigo Symptom Scale—Short Form, VSS: Vertigo Symptom Scale, PHQ: Patient Health Questionnaire, HADS: Hospital Anxiety and Depression Scale, GAD: Generalized Anxiety Disorder Assessment, BAI: Beck Inventory Scale, PETS: Patient Experience with Treatment and Self-management, VAS: Visual Analogue Scale, SF: Short Form, VR: Virtual Reality, GP: General Practitioner, QALY: Quality-Adjusted Life Years.

## Data Availability

Data are available upon reasonable request to the corresponding author.

## Supplementary Materials

The following supporting information can be downloaded at: https://www.mdpi.com/article/10.3390/s24103028/s1.

## References

[1] Ruthberg J.S., Rasendran C., Kocharyan A., Mowry S.E., Otteson T.D. (2021). The Economic Burden of Vertigo and Dizziness in the United States. J. Vestib. Res..

[2] Penger M., Strobl R., Grill E. (2017). Country-Specific and Individual Determinants of Dizziness in Europe: Results from the Survey of Health Ageing and Retirement in Europe (SHARE). Public Health.

[3] Murdin L., Schilder A.G.M. (2015). Epidemiology of Balance Symptoms and Disorders in the Community: A Systematic Review. Otol. Neurotol..

[4] Grill E., Akdal G., Becker-Bense S., Hübinger S., Huppert D., Kentala E., Strobl R., Zwergal A., Celebisoy N. (2018). Multicenter Data Banking in Management of Dizzy Patients: First Results from the DizzyNet Registry Project. J. Neurol..

[5] Grill E., Müller T., Becker-Bense S., Gürkov R., Heinen F., Huppert D., Zwergal A., Strobl R. (2017). DizzyReg: The Prospective Patient Registry of the German Center for Vertigo and Balance Disorders. J. Neurol..

[6] Bisdorff A.R., Staab J.P., Newman-Toker D.E. (2015). Overview of the International Classification of Vestibular Disorders. Neurol. Clin..

[7] Strupp M., Dlugaiczyk J., Ertl-Wagner B.B., Rujescu D., Westhofen M., Dieterich M. (2020). Vestibular Disorders. Dtsch. Ärztebl. Int..

[8] Pfieffer M.L., Anthamatten A., Glassford M. (2019). Assessment and Treatment of Dizziness and Vertigo. Nurse Pract..

[9] Drachman D.A., Hart C.W. (1972). An Approach to the Dizzy Patient. Neurology.

[10] Kerber K.A., Newman-Toker D.E. (2015). Misdiagnosing Dizzy Patients. Neurol. Clin..

[11] Karatas M. (2008). Central Vertigo and Dizziness: Epidemiology, Differential Diagnosis, and Common Causes. Neurologist.

[12] Seemungal B.M. (2022). The Bárány Society Position on ‘Cervical Dizziness’. J. Vestib. Res..

[13] Neuhauser H.K. (2016). Neuro-Otology: Diagnosis and Management of Neuro-Otological Disorders. Handbook of Clinical Neurology.

[14] Hall C.D., Herdman S.J.P., Whitney S.L.D., Anson E.R., Carender W.J.P., Hoppes C.W.P., Cass S.P., Christy J.B., Cohen H.S.O., Fife T.D.M. (2022). Vestibular Rehabilitation for Peripheral Vestibular Hypofunction: An Updated Clinical Practice Guideline from the Academy of Neurologic Physical Therapy of the American Physical Therapy Association. J. Neurol. Phys. Ther..

[15] Zhang S., Liu D., Tian E., Wang J., Guo Z., Kong W. (2022). Central Vestibular Dysfunction: Don’t Forget Vestibular Rehabilitation. Expert Rev. Neurother..

[16] Vestel C. (2022). Systematic Review and Meta-Analysis of the Therapeutic Management of Patients with Cervicogenic Dizziness. J. Man. Manip. Ther..

[17] Tramontano M., Russo V., Spitoni G.F., Ciancarelli I., Paolucci S., Manzari L., Morone G. (2021). Efficacy of Vestibular Rehabilitation in Patients with Neurologic Disorders: A Systematic Review. Arch. Phys. Med. Rehabil..

[18] Regauer V., Seckler E., Müller M., Bauer P. (2020). Physical Therapy Interventions for Older People with Vertigo, Dizziness and Balance Disorders Addressing Mobility and Participation: A Systematic Review. BMC Geriatr..

[19] Heydari M., Ahadi M., Jalaei B., Maarefvand M., Talebi H. (2021). The Additional Effect of Vestibular Rehabilitation Therapy on Residual Dizziness after Successful Modified Epley Procedure for Posterior Canal Benign Paroxysmal Positional Vertigo. Am. J. Audiol..

[20] Cooksey F.S. (1946). Rehabilitation in Vestibular Injuries. Proc. R. Soc. Med..

[21] Han B.I., Song H.S., Kim J.S. (2011). Vestibular Rehabilitation Therapy: Review of Indications, Mechanisms, and Key Exercises. J. Clin. Neurol..

[22] Klatt B.N. (2015). A Conceptual Framework for the Progression of Balance Exercises in Persons with Balance and Vestibular Disorders. Phys. Med. Rehabil. Int..

[23] Yu Y.-C., Xue H., Zhang Y., Zhou J. (2018). Cognitive Behavior Therapy as Augmentation for Sertraline in Treating Patients with Persistent Postural-Perceptual Dizziness. BioMed Res. Int..

[24] Herdman D., Norton S., Murdin L., Frost K., Pavlou M., Moss-Morris R. (2022). The INVEST Trial: A Randomised Feasibility Trial of Psychologically Informed Vestibular Rehabilitation versus Current Gold Standard Physiotherapy for People with Persistent Postural Perceptual Dizziness. J. Neurol..

[25] Shaver J. (2022). The State of Telehealth Before and After the COVID-19 Pandemic. Prim. Care Clin. Off. Pract..

[26] Zheng J., Hou M., Liu L., Wang X. (2022). Knowledge Structure and Emerging Trends of Telerehabilitation in Recent 20 Years: A Bibliometric Analysis via CiteSpace. Front. Public Health.

[27] Miller M.J., Pak S.S., Keller D.R., Gustavson A.M., Barnes D.E. (2022). Physical Therapist Telehealth Delivery at 1 Year Into COVID-19. Phys. Ther..

[28] Werneke M.W., Deutscher D., Grigsby D., Tucker C.A., Mioduski J.E., Hayes D. (2021). Telerehabilitation During the COVID-19 Pandemic in Outpatient Rehabilitation Settings: A Descriptive Study. Phys. Ther..

[29] Seron P., Oliveros M.-J., Gutierrez-Arias R., Fuentes-Aspe R., Torres-Castro R.C., Merino-Osorio C., Nahuelhual P., Inostroza J., Jalil Y., Solano R. (2021). Effectiveness of Telerehabilitation in Physical Therapy: A Rapid Overview. Phys. Ther..

[30] Kocyigit B.F., Assylbek M.I., Yessirkepov M. (2024). Telerehabilitation: Lessons from the COVID-19 Pandemic and Future Perspectives. Rheumatol. Int..

[31] Baroni M.P., Jacob M.F.A., Rios W.R., Fandim J.V., Fernandes L.G., Chaves P.I., Fioratti I., Saragiotto B.T. (2023). The State of the Art in Telerehabilitation for Musculoskeletal Conditions. Arch. Physiother..

[32] León-Salas B., González-Hernández Y., Infante-Ventura D., de Armas-Castellano A., García-García J., García-Hernández M., Carmona-Rodríguez M., Olazarán J., Dobato J.L., Rodríguez-Rodríguez L. (2023). Telemedicine for Neurological Diseases: A Systematic Review and Meta-Analysis. Eur. J. Neurol..

[33] Calabrò R.S., Bonanno M., Torregrossa W., Cacciante L., Celesti A., Rifici C., Tonin P., De Luca R., Quartarone A. (2023). Benefits of Telerehabilitation for Patients with Severe Acquired Brain Injury: Promising Results from a Multicenter Randomized Controlled Trial Using Nonimmersive Virtual Reality. J. Med. Internet Res..

[34] Shem K., Irgens I., Alexander M.G.S. (2022). Mechanisms of Telerehabilitation. Telerehabilitation 5–20.

[35] Cottrell M.A., Russell T.G. (2020). Telehealth for Musculoskeletal Physiotherapy. Musculoskelet. Sci. Pract..

[36] Green K.E., Pogson J.M., Otero-Millan J., Gold D.R., Tevzadze N., Tehrani A.S.S., Zee D.S., Newman-Toker D.E., Kheradmand A. (2021). Opinion and Special Articles: Remote Evaluation of Acute Vertigo: Strategies and Technological Considerations. Neurology.

[37] Shaikh A.G., Bronstein A., Carmona S., Cha Y.-H., Cho C., Ghasia F.F., Gold D., Green K.E., Helmchen C., Ibitoye R.T. (2021). Consensus on Virtual Management of Vestibular Disorders: Urgent Versus Expedited Care. Cerebellum.

[38] Schoo D.P., Ward B.K. (2021). New Frontiers in Managing the Dizzy Patient. Otolaryngol. Clin. N. Am..

[39] Bertholon P., Thai-Van H., Bouccara D., Esteve-Fraysse M.-J., Wiener-Vacher S., Ionescu E. (2021). Guidelines of the French Society of Otorhinolaryngology (SFORL) for Teleconsultation in Patients with Vertigo during the COVID-19 Pandemic. Eur. Ann. Otorhinolaryngol. Head Neck Dis..

[40] Harrell R.G., Schubert M.C., Oxborough S., Whitney S.L. (2022). Vestibular Rehabilitation Telehealth During the SAEA-CoV-2 (COVID-19) Pandemic. Front. Neurol..

[41] Meldrum D., Murray D., Vance R., Coleman S., McConnell S., Hardiman O., Walsh R.M. (2022). Toward a Digital Health Intervention for Vestibular Rehabilitation: Usability and Subjective Outcomes of a Novel Platform. Front. Neurol..

[42] Page M.J., McKenzie J.E., Bossuyt P.M., Boutron I., Hoffmann T.C., Mulrow C.D., Shamseer L., Tetzlaff J.M., Akl E.A., Brennan S.E. (2021). The PRISMA 2020 Statement: An Updated Guideline for Reporting Systematic Reviews. BMJ.

[43] Schiavenato M., Chu F. (2021). PICO: What It Is and What It Is Not. Nurse Educ. Pract..

[44] Yardley L., Beech S., Zander L., Evans T., Weinman J. (1998). A Randomized Controlled Trial of Exercise Therapy for Dizziness and Vertigo in Primary Care. Br. J. Gen. Pract..

[45] Söderman A.-C.H., Bergenius J., Bagger-Sjöbäck D., Tjell C., Langius A. (2001). Patients’ Subjective Evaluations of Quality of Life Related to Disease-Specific Symptoms, Sense of Coherence, and Treatment in MéNièRe’s Disease. Otol. Neurotol..

[46] Jacobson G.P., Newman C.W. (1990). The Development of the Dizziness Handicap Inventory. Arch. Otolaryngol.-Head Neck Surg..

[47] Kroenke K., Spitzer R.L., Williams J.B. (2001). The PHQ-9: Validity of a Brief Depression Severity Measure. J. Gen. Intern. Med..

[48] Zigmond A.S., Snaith R.P. (1983). The Hospital Anxiety and Depression Scale. Acta Psychiatr. Scand..

[49] Bjelland I., Dahl A.A., Haug T.T., Neckelmann D. (2002). The Validity of the Hospital Anxiety and Depression Scale. J. Psychosom. Res..

[50] Piker E.G., Kaylie D.M., Garrison D., Tucci D.L. (2015). Hospital Anxiety and Depression Scale: Factor Structure, Internal Consistency and Convergent Validity in Patients with Dizziness. Audiol. Neurotol..

[51] Löwe B., Decker O., Müller S., Brähler E., Schellberg D., Herzog W., Herzberg P.Y. (2008). Validation and Standardization of the Generalized Anxiety Disorder Screener (GAD-7) in the General Population. Med. Care.

[52] Kjærgaard M., Arfwedson Wang C.E., Waterloo K., Jorde R. (2014). A Study of the Psychometric Properties of the Beck Depression Inventory-II, the Montgomery and Åsberg Depression Rating Scale, and the Hospital Anxiety and Depression Scale in a Sample from a Healthy Population. Scand. J. Psychol..

[53] Wang Y.-P., Gorenstein C. (2013). Psychometric Properties of the Beck Depression Inventory-II: A Comprehensive Review. Rev. Bras. Psiquiatr..

[54] Fong E., Li C., Aslakson R., Agrawal Y. (2015). Systematic Review of Patient-Reported Outcome Measures in Clinical Vestibular Research. Arch. Phys. Med. Rehabil..

[55] Feng S., Zang J. (2023). The Effect of Accompanying Anxiety and Depression on Patients with Different Vestibular Syndromes. Front. Aging Neurosci..

[56] Radziej K., Probst T., Limburg K., Dinkel A., Dieterich M., Lahmann C. (2018). The Longitudinal Effect of Vertigo and Dizziness Symptoms on Psychological Distress: Symptom-Related Fears and Beliefs as Mediators. J. Nerv. Ment. Dis..

[57] Prell T., Axer H. (2022). Avoidance Behavior in Patients with Chronic Dizziness: A Prospective Observational Study. J. Clin. Med..

[58] Omara A., Basiouny E.M., Shabrawy M.E., Shafei R.R.E. (2022). The Correlation between Anxiety, Depression, and Vertigo: A Cross-Sectional Study. Egypt. J. Otolaryngol..

[59] Rizk H.G., Liu Y.F. (2021). Interviewing and Counseling the Dizzy Patient with Focus on Quality of Life. Otolaryngol. Clin. N. Am..

[60] Ciorba A., Bianchini C., Scanelli G., Pala M., Zurlo A., Aimoni C. (2017). The Impact of Dizziness on Quality-of-Life in the Elderly. Eur. Arch. Otorhinolaryngol..

[61] Weidt S., Bruehl A.B., Straumann D., Hegemann S.C., Krautstrunk G., Rufer M. (2014). Health-Related Quality of Life and Emotional Distress in Patients with Dizziness: A Cross-Sectional Approach to Disentangle Their Relationship. BMC Health Serv. Res..

[62] Sterne J.A.C., Savović J., Page M.J., Elbers R.G., Blencowe N.S., Boutron I., Cates C.J., Cheng H.Y., Corbett M.S., Eldridge S.M. (2019). RoB 2: A revised tool for assessing risk of bias in randomised trials. BMJ.

[63] Collaboration T.C. (2020). Review Manager (RevMan), 5.4.

[64] Spitzer R.L., Kroenke K., Williams J.B.W., Löwe B. (2006). A Brief Measure for Assessing Generalized Anxiety Disorder: The GAD-7. Arch. Intern. Med..

[65] Higgins J.P.T., Thomas J., Chandler J., Cumpston M., Li T., Page M.J., Welch V.A. (2019). Front Matter. Cochrane Handbook for Systematic Reviews of Interventions.

[66] Szturm T., Hochman J., Wu C., Lisa L., Reimer K., Wonneck B., Giacobbo A. (2015). Games and Telerehabilitation for Balance Impairments and Gaze Dysfunction: Protocol of a Randomized Controlled Trial. JMIR Res. Protoc..

[67] Geraghty A.W.A., Essery R., Kirby S., Stuart B., Turner D., Little P., Bronstein A., Andersson G., Carlbring P., Yardley L. (2017). Internet-Based Vestibular Rehabilitation for Older Adults with Chronic Dizziness: A Randomized Controlled Trial in Primary Care. Ann. Fam. Med..

[68] Vugt V.A., Wouden J.C., Essery R., Yardley L., Twisk J.W.R., Horst H.E., Maarsingh O. (2019). Internet Based Vestibular Rehabilitation with and without Physiotherapy Support for Adults Aged 50 and Older with a Chronic Vestibular Syndrome in General Practice: Three Armed Randomised Controlled Trial. BMJ.

[69] Yardley L., Barker F., Muller I., Turner D., Kirby S., Mullee M., Morris A., Little P. (2012). Clinical and cost effectiveness of booklet based vestibular rehabilitation for chronic dizziness in primary care: Single blind, parallel group, pragmatic, randomised controlled trial. BMJ.

[70] Haciabbasoğlu R., Araci A., Günizi H. (2023). Are Telerehabilitation Exercise Practices Effective in Patients Diagnosed with Benign Paroxysmal Positional Vertigo?. Indian J. Otolaryngol. Head Neck Surg..

[71] Smaerup M., Grönvall E., Larsen S.B., Laessoe U., Henriksen J.-J., Damsgaard E.M. (2015). Computer-Assisted Training as a Complement in Rehabilitation of Patients with Chronic Vestibular Dizziness—A Randomized Controlled Trial. Arch. Phys. Med. Rehabil..

[72] Smaerup M., Laessoe U., Grönvall E., Henriksen J.-J., Damsgaard E.M. (2016). The Use of Computer-Assisted Home Exercises to Preserve Physical Function after a Vestibular Rehabilitation Program: A Randomized Controlled Study. Rehabil. Res. Pract..

[73] Yardley L., Donovan-Hall M., Smith H.E., Walsh B.M., Mullee M., Bronstein A.M. (2004). Effectiveness of Primary Care–Based Vestibular Rehabilitation for Chronic Dizziness. Ann. Intern. Med..

[74] Essery R., Kirby S., Geraghty A.W.A., Andersson G., Carlbring P., Bronstein A., Little P., Yardley L. (2015). The Development of Balance Retraining: An Online Intervention for Dizziness in Adults Aged 50 Years and Older. Am. J. Audiol..

[75] Boyd R.N. (2013). Move It to Improve It (Mitii): Study Protocol of a Randomised Controlled Trial of a Novel Web-Based Multimodal Training Program for Children and Adolescents with Cerebral Palsy. BMJ Open.

[76] Beukes E.W., Manchaiah V., Allen P.M., Baguley D.M., Andersson G. (2019). Internet-Based Interventions for Adults with Hearing Loss, Tinnitus, and Vestibular Disorders: A Systematic Review and Meta-Analysis. Trends Hear..

[77] Webster K.E., Kamo T., Smith L., Harrington-Benton N.A., Judd O., Kaski D., Maarsingh O.R., MacKeith S., Ray J., Vugt V.A. (2023). Non-pharmacological Interventions for Persistent Postural-perceptual Dizziness (PPPD). Cochrane Database Syst. Rev..

[78] Chu H.-Y., Song N., Zhou Z.-R., Li Z.-F., Yang X. (2023). Can Virtual Reality-Assisted Therapy Offer Additional Benefits to Patients with Vestibular Disorders Compared with Conventional Vestibular Physical Therapy? A Meta-Analysis. Arch. Phys. Med. Rehabil..

[79] Suso-Martí L., La Touche R., Herranz-Gómez A., Angulo-Díaz-Parreño S., Paris-Alemany A., Cuenca-Martínez F. (2021). Effectiveness of Telerehabilitation in Physical Therapist Practice: An Umbrella and Mapping Review with Meta–Meta-Analysis. Phys. Ther..

[80] Dias J.F., Oliveira V.C., Borges P.R.T., Dutra F.C.M.S., Mancini M.C., Kirkwood R.N., Resende R.A., Sampaio R.F. (2021). Effectiveness of Exercises by Telerehabilitation on Pain, Physical Function and Quality of Life in People with Physical Disabilities: A Systematic Review of Randomised Controlled Trials with GRADE Recommendations. Br. J. Sports Med..

[81] Gaikwad S.B., Mukherjee T., Shah P.V., Ambode O.I., Johnson E.G., Daher N.S. (2016). Home Exercise Program Adherence Strategies in Vestibular Rehabilitation: A Systematic Review. Phys. Ther. Rehabil. Sci..

[82] Meldrum D., Burrows L., Cakrt O., Kerkeni H., Lopez C., Tjernstrom F., Vereeck L., Zur O., Jahn K. (2020). Vestibular rehabilitation in Europe: A survey of clinical and research practice. J. Neurol..

[83] Muller I., Kirby S., Yardley L. (2015). Understanding Patient Experiences of Self-Managing Chronic Dizziness: A Qualitative Study of Booklet-Based Vestibular Rehabilitation, with or without Remote Support. BMJ Open.

[84] Eldøen G., Kvalheim S.E., Thesen T., Mygland Å., Ljøstad U., Bakke S., Holo M.H., Løge I., Jonsbu E. (2021). Web-based Vestibular Rehabilitation in Persistent Postural-perceptual Dizziness. Brain Behav..

[85] Hovareshti P., Roeder S., Holt L.S., Gao P., Xiao L., Zalkin C., Ou V., Tolani D., Klatt B.N., Whitney S.L. (2021). VestAid: A Tablet-Based Technology for Objective Exercise Monitoring in Vestibular Rehabilitation. Sensors.

[86] Beh S.C. (2021). The Neuropsychology of Dizziness and Related Disorders. Otolaryngol. Clin. N. Am..

[87] Carender W.J., Grzesiak M., Telian S.A. (2021). Vestibular Physical Therapy and Fall Risk Assessment. Otolaryngol. Clin. N. Am..

[88] Hülse R., Biesdorf A., Hörmann K., Stuck B., Erhart M., Hülse M., Wenzel A. (2019). Peripheral Vestibular Disorders: An Epidemiologic Survey in 70 Million Individuals. Otol. Neurotol..

[89] McDonnell M.N., Hillier S.L. (2015). Vestibular Rehabilitation for Unilateral Peripheral Vestibular Dysfunction. Cochrane Database Syst. Rev..

