# Supplementary material for: Effectiveness of Telerehabilitation in Dizziness: A Systematic Review with Meta-Analysis
Source: Sensors (Basel). 2024 May 10;24(10):3028. doi: 10.3390/s24103028 (PMC11125243; doi:10.3390/s24103028)
Supplement: Supplementary file 1 [file sensors-24-03028-s001.zip › sensors-2939252-supplementary.pdf]

Supplementary Appendix SA

PubMed research string:

("benign paroxysmal positional vertigo/nursing"[MeSH Terms] OR "benign paroxysmal positional vertigo/rehabilitation"[MeSH Terms] OR "vertigo/nursing"[MeSH Terms] OR "vertigo/rehabilitation"[MeSH Terms] OR "vertigo/therapy"[MeSH Terms] OR "labyrinth diseases/nursing"[MeSH Terms] OR "labyrinth diseases/rehabilitation"[MeSH Terms] OR "labyrinth diseases/therapy"[MeSH Terms] OR "vestibular diseases/nursing"[MeSH Terms] OR "vestibular diseases/rehabilitation"[MeSH Terms] OR "vestibular diseases/therapy"[MeSH Terms] OR "dizziness/nursing"[MeSH Terms] OR"dizziness/rehabilitation"[MeSHTerms] OR "dizziness/therapy"[MeSH Terms] OR "acute peripheral vestibulopathies"[All Fields] OR "acute peripheral vestibulopathy"[All Fields] OR "acute vestibular neuritis"[All Fields] OR "acute vestibular neuritis"[All Fields] OR "vestibular syndrome"[All Fields] OR "vestibular disorders"[All Fields] OR "chronic vestibular dysfunction"[All Fields] OR "vestibular balance disorders"[All Fields] OR "chronic vestibular insufficiency"[All Fields] OR "Benign Paroxysmal Positional Vertigo"[MeSH Terms] OR "meniere disease"[MeSH Terms]) OR "Benign Paroxysmal Positional Vertigo"[All Fields] OR "Peripheral vestibular dysfunction"[All Fields] OR "Vestibular migraine"[All Fields] OR "Meniere's disease"[All Fields] OR "Meniere's syndrome"[All Fields] OR "Benign Paroxysmal Positional Vertigo"[All Fields] OR "BPPV"[All Fields] OR "Vestibular nerve"[All Fields] OR "endolymphatic hydrops"[MeSH Terms] OR "endolymphatic hydrops"[All Fields] OR "cervicogenic dizziness" OR "cervical vertigo" OR "dizziness" OR "vertigo" OR (("Dizziness/rehabilitation"[Mesh]) OR ("Dizziness/therapy"[Mesh])) OR ("Vertigo/rehabilitation"[Mesh] OR "Vertigo/therapy"[Mesh]) AND ("telerehab*"[All Fields] OR "tele rehab*"[All Fields] OR "telemed*"[All Fields] OR "tele med*"[All Fields] OR "telehealth*"[All Fields] OR "tele health*"[All Fields] OR "teleconsult*"[All Fields] OR "tele consult*"[All Fields] OR "teleconf*"[All Fields] OR "tele conf*"[All Fields] OR "telehom*"[All Fields] OR "tele hom*"[All Fields] OR "telecoach*"[All Fields] OR "tele coach*"[All Fields] OR "telecare*"[All Fields] OR "tele care*"[All Fields] OR "teletherap*"[All Fields] OR "tele therap*"[All Fields] OR "erehab*"[All Fields] OR "e rehab*"[All Fields] OR "ehealth*"[All Fields] OR "e health*"[All Fields] OR "emed*"[All Fields] OR "e med*"[All Fields] OR "virtual rehab*"[All Fields] OR "videoconf*"[All Fields] OR "technology based*"[All Fields] OR "internet based*"[All Fields] OR "remote*"[All Fields] OR "telemedicine"[MeSH Terms] OR "telemetry"[MeSH Terms] OR "videoconferencing"[MeSH Terms] OR "user computer interface"[MeSH Terms] OR "Telerehabilitation"[MeSH Terms])

Cochrane research string:

"vestibular syndrome" OR "vestibular disorders" OR "labirinth diseases" OR " acute peripheral vestibulopat*" OR "acute peripheral vestibulopat*" OR "acute vestibular neuritis" OR "chronic vestibular dysfunction" OR " vestibular balance disorder*" OR "chronic vestibular insufficiency" OR "benignal paroxysmal positional vertigo" OR "BPPV" OR "peripheral vestibular dysfunction" OR "vestibular migraine" OR "Meniere's disease" OR "meniere's syndrome" OR "cervical dizziness" OR "cervical vertigo" OR "dizziness" OR "vertigo" OR MeSH descriptor: [Dizziness] explode all trees OR MeSH descriptor: [Vestibular Diseases] explode all trees AND "telerehab*" OR "tele rehab*" OR "telemed*" OR "tele med*" OR "telehealth*" OR "tele health*" OR "teleconsult*" OR "tele consult*" OR "teleconf*" OR "tele conf*" OR "telehom*" OR "tele hom*" OR "telecoach*" OR "tele coach*" OR "telecare*" OR "tele care*" OR "teletherap*" OR "teletherap*" OR "erehab*" OR "e rehab*" OR "ehealth*" OR "e health*" OR

"emed*" OR "e med*" OR "virtual rehab*" OR "videoconf*" OR "technology based*" OR "internet based*" OR "remote*" OR "telemedicine" OR "telemetry" OR "videoconferencing" OR "user computer interface" OR "Telerehabilitation" OR MeSH descriptor: [Telerehabilitation] explode all trees

Pedro research string: free terms:

- Tele rehabilitation
- Tele
- Vestibular disorders
- Dizziness
- Vertigo

Supplementary Appendix SB

Table S1. Study eligibility criteria.

Inclusion	Exclusion
Population<br>• Adult participants (age ≥18 years) with vertigo symptoms of various etiologies, regardless the timing of symptoms (acute or chronic), that received rehabilitation treatment through tele-rehabilitation.<br>Etiology can include central, peripheral, nonspecific, cervicogenic causes.	
Interventions<br>• Rehabilitation treatments through tele-rehabilitation provided remotely, both synchronous and asynchronous, which include:<br>- Remote rehabilitation sessions with a therapist.<br>- Tele-consultation sessions with a therapist and individual treatment independently of the patient.<br>- Alternating tele-consultation/remote rehabilitation sessions and presence sessions. Monitoring through tele-consultation, presence rehabilitation treatment.<br>- Remote consultation/rehabilitation sessions and other forms of treatment (face to face, internet-based interventions, digital platforms or software or applications).<br>- Remote asynchronous teleconsultation/rehabilitation sessions (internet-based interventions, digital platforms or software or applications).	• rehabilitation treatments carried autonomously and remotely by patient, even through technologies (i.e. digital platform, website etc), but no performance data is provided or displayed by the therapist through information and communication technologies (no synchronous or asynchronous data sharing).

Control<br>• Any other treatment not delivered through telerehabilitation, such as:<br>- In presence rehabilitation alone;<br>- Remote therapy sessions without any data exchange through information and communication technologies;<br>- Usual care (education, pharmacologic support, etc).<br><br>• No treatment	
Outcomes<br>• Primary:<br>- reduction of vertigo symptoms<br>• Secondary:<br>- improvement of quality of life, improvement of psychological impairments..	
Study design<br>• randomised controlled trial (RCT)	• Other designs: systematic reviews, cohort trials, case trials/ case series
Publication type<br>• full-text study publications	
Publication Period<br>• no limits	
Language<br>• english, italian	

Table S2. List of xcluded studies.

**Title**	**Year**	**Study design**	**Exclusion reason**
Effect of Self-treatment of Recurrent Benign Paroxysmal Positional Vertigo: A randomized clinical trial.	2023	RCT	Full text not available
Home-based Vestibular Rehabilitation: A Feasible and Effective Therapy for Persistent Postural Perceptual Dizziness.	2023	PILOT RCT	Not Telerehabilitatio n treatment
Effectiveness of conventional versus virtual reality-based vestibular rehabilitation exercises in elderly patients with dizziness: a randomized controlled study with 6-month follow-up.	2022	RCT	Not Telerehabilitatio n treatment
Comparison of Activity-Based Home Program an Cawthorne-Cooksey Exercises in Patients With Chronic Unilateral Peripheral Vestibular Disorders.	2021	RCT	Not Telerehabilitatio n treatment

Vestibular rehabilitation in older adults with and without mild cognitive impairment: Effects of virtual reality using a head-mounted display.	2019	RCT	Not Telerehabilitation treatment
Vestibular rehabilitation using video gaming in adults with dizziness: a pilot study	2018	PILOT RCT	Not Telerehabilitation treatment
The efficacy of a home treatment program combined with office-based canalith repositioning procedure for benign paroxysmal positional vertigo: a randomized controlled trial.	2019	RCT	Full text not available
Reducing the burden of dizziness in middleaged and older people: A multifactorial, tailored, single-blind randomized controlled trial.	2018	RCT	CBT Telerehabilitation treatment
Web-Based Telepresence Exercise Program for Community-Dwelling Elderly Women With a High Risk of Falling: Randomized Controlled Trial.	2018	RCT	Consider different populations
Internet-Based Self-Help for Ménière's Disease: Details and Outcome of a Single-Group Open Trial.	2017	RCT	Full text not available
Three-dimensional head-mounted gaming task procedure maximizes effects of vestibular rehabilitation in unilateralvestibular hypofunction: a randomized controlled pilot trial.	2017	PILOT RCT	Not Telerehabilitation treatment
ICT-based system to predict and prevent falls (iStoppFalls): results from an international multicenter randomized controlled trial.	2015	RCT	Consider different populations
Home-Based Computer Gaming in Vestibular Rehabilitation of Gaze and Balance Impairment	2015	Case series	Study design not eligible
Evaluation of Booklet-Based Self-Management of Symptoms in Meniere Disease: A Randomized Controlled Trial	2006	RCT	Not Telerehabilitation treatment
A randomized controlled trial of exercise therapy for dizziness and vertigo in primary care	1998	RCT	Not Telerehabilitation treatment
